# Impact socio professionnel de la libération chirurgicale du syndrome du canal carpien

**DOI:** 10.11604/pamj.2016.24.234.9259

**Published:** 2016-07-13

**Authors:** Aouatef Mahfoudh Kraiem, Hajer Hnia, Lamia Bouzgarrou, Mohamed Adnène Henchi, Taoufik Khalfallah

**Affiliations:** 1 Département de Médecine de Travail et d’Ergonomie-Faculté de Médecine de Monastir, Tunisie

**Keywords:** Syndrome de canal carpien, traitement chirurgical, devenir professionnel, Carpal tunnel syndrome, surgical treatment, professional future

## Abstract

L’objectif de notre travail était d’étudier les conséquences socioprofessionnelles d’une libération chirurgicale du SCC. Il s’agit d’une étude transversale portant sur les sujets opérés pour un SCC d’origine professionnelle ; recensés dans le Service de Médecine de Travail et de Pathologies Professionnelles au CHU Tahar Sfar de Mahdia en Tunisie sur une période de 8 ans allant du 1 Janvier 2006 au mois Décembre 2013. Le recueil des données s’est basé sur une fiche d’enquête, portant sur la description des caractéristiques socioprofessionnelles, médicales, et sur le devenir professionnel des participants. Pour étudier les contraintes psychosociales au travail, nous avons adopté le questionnaire de Karasek. La durée d’arrêt de travail après libération chirurgicale du SCC était significativement liée à l’existence d’autres troubles musculo-squelettiques autre que le SCC, la déclaration du SCC en maladie professionnelle et à l’ancienneté professionnelle des salariés. Quant au devenir professionnel des salariés opérés, 50,7% ont gardé le même poste, 15,3% ont bénéficié d’un aménagement de poste et 33,8% ont bénéficié d’un changement de poste dans la même entreprise. Le devenir professionnel de ces salariés était corrélé à leurs qualifications professionnelles et au type de l’atteinte sensitive et/ou motrice du nerf médian à l’EMG. Un certain nombre de facteurs non lésionnels déterminaient la durée de l’arrêt de travail, alors que le devenir professionnel des opérés pour SCC dépendait essentiellement de leurs qualifications professionnelles et des données de l’électromyogramme. Il est certain que des travaux beaucoup plus larges permettraient d’affiner encore ces résultats.

## Abstract (en)

The objective was studying the socio-professional impact of release surgery for carpal tunnel syndrom (CTS). We conducted a cross-sectional study of patients operated for work-related CTS; data were collected in the Occupational Health Department at the University Hospital Tahar Sfar in Mahdia, Tunisia over a period of 8 years, from 1 January 2006 to December 2013. Data collection was performed using a survey form focusing on participants’ socio-professional and medical characteristics and on their professional future. We used Karasek’s questionnaire to study psychosocial constraints at work. The duration of a work stoppage following release surgery for CTS was significantly related to the existence of musculoskeletal disorders other than CTS, to a statement that the carpal tunnel syndrome was work related and to job seniority. As regards the professional future of operated employees, 50.7% remained in the same position, 15.3% were given customized workstation and 33.8% were offered a different position within the same company. The professional future of these employees was related to their occupational qualifications and to the type of sensory and/or motor impairment of median nerve detected during EMG test. A number of nonlesional factors determines the duration of the work stoppage, while the professional future of patients operated for CTS essentially depends on their professional qualifications and on EMG data. Certainly much broader studies would allow to refine these results.

## Introduction

Le retour au travail des patients opérés pour syndrome du canal carpien est un processus complexe et multifactoriel. Dans la littérature, la durée d’interruption professionnelle est un événement communément utilisé pour comparer les méthodes thérapeutiques du SCC. Les critères objectifs sont rarement décrits pour évaluer cet événement. Ainsi, et dans la grande majorité des études examinant l’apport de la libération chirurgical du canal carpien ainsi que le traitement des fractures du scaphoïde non déplacées dans le retour au travail, relevaient des critères vagues, subjectifs, voire inexistants [1]. Le chirurgien se trouve souvent influencer par des facteurs subjectifs liés au patient lui-même (la douleur, la motivation, des idées préconçues, etc …). Certains auteurs avaient incriminés le phénomène de « comportement d’évitement de peur » dans lequel les croyances sur la douleur sont associées aux degrés d´inquiétude et à la peur [2, 3]. Ces schémas cognitifs peuvent influencés l’interprétation de l´importance clinique de la douleur du patient et peut, par conséquent, influencer la décision médicale de mise en repos ou le retour au travail. Le médecin est considéré par la société comme l’arbitre d’une autorisation médicale pour le devoir professionnel, il doit se disposer des critères clairs et objectifs pour juger la période de cette autorisation. C’est dans ce cadre que nous avons mené ce travail dont les objectifs étaient d’étudier les conséquences socioprofessionnelles d’une libération chirurgicale du SCC.

## Méthodes

**Présentation de l’étude:** Il s’agit d’une étude descriptive rétrospective portant sur les sujets atteints de SCC d’origine professionnelle recensés dans le Service de Médecine de Travail et de Pathologies Professionnelles au CHU de Mahdia, en Tunisie sur une période de 9 ans allant du 1^er^ Janvier 2006 au 31 Décembre 2013.

**Population d’étude:** Ils ont été inclus dans cette étude, les patients d’âge compris entre 26 et 61 ans, en activité professionnelle et/ou en arrêt de travail à cause du SCC et ayant bénéficiés d’une libération chirurgicale du SCC. Selon la durée d’absence post-opératoire, la population d’étude a été subdivisée en deux groupes ((groupe 1 <8 semaines> groupe 2).

**Matériels:** Le recueil des informations s’est basé sur une fiche d’enquête préalablement élaborée et remplie par un médecin enquêteur. Cette fiche comporte la description des caractéristiques sociodémographiques (genre, âge, situation matrimonial, nombre d’enfant à charge…..), médicales (durée d’évolution des symptômes, signes fonctionnels, données cliniques….), et professionnelles (secteurs d’activité, ancienneté professionnelle, poste de travail…). Pour étudier les contraintes psychosociales au travail, nous avons adopté le questionnaire de Karasek. Ce modèle permet de faire un lien entre le vécu du travail et les risques que ce travail fait courir à la santé [4]. L’évaluation des conséquences du SCC a porté sur la détermination de la durée d’arrêt du travail en postopératoire et sur le devenir professionnel des patients (aménagement du poste, changement du poste, changement d’entreprise…).

**Etudes statistique:** Les moyennes, les médianes et les écarts types avec détermination de l’étendue (valeurs extrêmes) ont été rapportées pour les variables quantitatives. Pour l’étude uni variée, nous avons utilisé le test « t » de Student et le test « f » d’ANOVA pour la comparaison des moyennes. Nous avon utilisé le test Khi-deux pour la comparaison des variables qualitatives et dans certains cas, nous avons employé le test exact de Fisher. Pour étudier les facteurs qui influencent la durée de repos postopératoire et le devenir professionnel des patients, nous avons eu recours à une régression logistique binaire multiple. L’inclusion des variables indépendantes dans les modèles de régression était faite lorsque leur degré de signification était inférieur à 0,2. Pour les tests statistiques, le seuil de signification p a été fixé à 0,05.

## Résultats

### Description générale de la population étudiée

Au cours de la période d’étude, 65 dossiers ont été recensés. La population étudiée était à prédominance féminine (sexe ratio=0.05) avec une moyenne d’âge de 43.74 ± 8.1 ans. La plupart des patients était droitier (94.6%). Le secteur d’activité le plus représenté était celui de la confection (936% des cas). L’ancienneté professionnelle moyenne a été de 20.8 ± 6.2 ans. Au-delà de la moitié de la population d’étude était qualifiée (70.2%). Les principales contraintes biomécaniques rapportées étaient les mouvements répétitifs du poignet (92.2%) et le travail en force (41.4%). Une situation de job strain a été décrite par tous les patients. La durée moyenne d’arrêt de travail après libération chirurgicale du nerf médian a été de 4 ±2.9 mois. Le reclassement professionnel a été rapporté par 32 patients (49.3%) (Tableau 1).

**Table 1:** Répartition des patients opérés pour SCC selon le devenir professionnel

Devenir professionnel	Effectif des salariés opérés	
	*Nombre*	*%*
Même poste	33	50.7
Changement de poste dans la même entreprise	22	33.84
Changement d’entreprise	0	0
Aménagement du poste	*10*	15.3
Total	65	100

### Déterminants du retour au travail en post opératoire du SCC

La durée de repos post-opératoire d’une libération chirurgicale du SCC était corrélée significativement à une atteinte droite du nerf médian (p=0.01), à l’ancienneté au poste (0.04), à une exposition professionnelle à des travaux sollicitant l’appui carpien ou la pression prolongée sur le talon de la main et à la date de déclaration du SCC en maladie professionnelle (p=0,04) (Tableau 2). Au terme de la régression logistique, la durée d’arrêt du travail après libération chirurgicale du SCC était significativement liée à l’existence d’autres TMS, la date de déclaration du SCC en maladie professionnelle et à l’ancienneté professionnelle des patients (Tableau 3).

**Tableau 2:** Déterminants socioprofessionnels de la durée d’absence post-opératoire du SCC en étude uni variée

Caractéristiques socioprofessionnelles	Durée d’absence post-opératoire de SCC	P
	0-8 semaines		>9 semaines
***Genre***			***1***
Femme	16 (24.6%)	47 (72.3%)	
Homme	0	2 (3.1)%	
***Age (ans)***			**0.50**
< 40 ans	9 (13.8%)	20 (30.6%)	
≥40 ans	7 (10.6%)	29 (44.5%)	
***Etat civil***			**0.22**
Marié (e)	8 (12.3%)	34 (52.3%)	
Célibataire	4 (6.1%)	7(10.7%)	
Autres	4 (6.1%)	8 (12.2%)	
***Autres pathologies musculosquelettiques***	14 (21.5%	31 (29.2%)	**0.11**
***Membre atteint***			**0.08**
Unilatéral	6 (9.2%)	11 (16.9%)	
Bilatéral	10 (15.3%)	38 (58.4%)	
***Données de l’éléctromyogramme***			
Côté atteint	-	-	**0.01**
Type de l’atteinte à droite	-	-	**0.50**
Type de l’atteinte à gauche	-	-	**0.47**
***Secteur d’activité***			**1**
Confection-textile	12 (18.4%)	38 (58.4%)	
Autre secteur	4 (6.1%)	11 (1.5%)	
***Ancienneté professionnelle***			**0.04**
< 10 ans	3 (4.6%)	1 (1.5%)	
≥10 ans	13 (20.2%)	48 (73.6%)	
***Qualification***			**0.42**
Quailifié	15 (23%)	40 (61.5%)	
Non qualifié	1 (1.5%)	9 (13.8%)	
***Contraintes biomécaniques liées au travail***			
Travail en force	7 (10.7%)	20 (30.7%)	**1**
Tâches répétitives du poignet	16 (24.6%)	44 (67.6%)	**0.32**
Appui carpien ou pression sur le talon de la main	2(3%)	0	**0.05**
***Contraintes psychosociales***			
Demande psychologique	23,5±1,59	23,2±2,12	**0.19**
Latitude décisionnelle	55,2±4,55	55,1±5,08	**0.81**
Soutien social	18,68±2,72	18,61±1,81	**0.10**
***Conséquences médico-légales***			
Déclaration du SCC en maladie professionnelle (MP )	16 (24.6%)	49 (75.3%)	**0.04**
Reconnaissance du SCC en MP	14 (24.5%)	39 (60%)	**0.65**

**Table 3:** Facteurs influençant la durée d’arrêt de travail en post opératoire du SCC après régression logistique multiple

	p	OR	IC
Autres TMS	0.036	0.12	[0.01-0.87]
Ancienneté professionnelle	0.038	3.05	[1.06-8.76]
Déclaration du SCC en maladie professionnelle	0.033	0.04	[0.03-0.77]

### Devenir professionnel des salariés opérés pour un SCC

Dans notre étude, aucun facteur sociodémographique ni professionnel n’influençait le devenir professionnel des salariés opérés du SCC en étude uni variée (Tableau 4). Au terme de l’analyse multivariée, le devenir professionnel des salariés opérés pour SCC était significativement corrélé à la qualification professionnelle (p=0.05) et au type de l’atteinte sensitive et/ou motrice du nerf médian à l’EMG (p=0.05) (Tableau 5).

**Table 4:** Déterminants socioprofessionnels du reclassement professionnel des opérés pour SCC en étude uni variée

Caractéristiques socioprofessionnelles	Reclassement professionnel	p
	Non		Oui
***Genre***			**1**
Femmes	32(49,23%)	31(47,6%)	
Hommes	1(1,5%)	1(1,5%)	
***Age (ans)***			**0.28**
<40	11(16,8%)	18(27.6%)	
≥40	22(33.8%)	14(21.4%)	
***Autres pathologies musculo-squelettiques***	24(36,9%)	21(32.3%)	**0.42**
***Membreatteint***			**0.81**
Unilatéral	8(12,3%)	9(13,8%)	
Bilatéral	25(38,4%)	23(35,3%)	
***Electromyogramme***	7.1%	33.3%	
Côtéatteint	-	-	**0.76**
Type de l’atteinte à droite	-	-	**0.08**
Type de l’atteinte à gauche	-	-	**0.32**
***Secteur activité***			**0.24**
Confection-textile	23(35,3%)	27(41,5%)	
Autresecteur	10(15,3%)	5(7 ,6%)	
***Poste occupé***			**0.20**
Ouvrière sur machine	17(26,15%)	22(33,8%)	
Autre	16(24,6%)	10(15,3%)	
***Ancienneté professionnelle***	0%	0%	**0.36**
<10	2(3%)	2(3%)	
>10	31(26,5%)	30(25,0%)	
***Qualification***			**0.08**
Qualifié	25(38,4%)	30(46,15%)	
Non qualifié	8(12,3%)	2(3%)	
***Contraintes biomécaniques***	0%	0%	**0.61**
Travail en force	0%12(18,4%)	15(23%)	
Travaux à tâches répétitives du poignet	30(46,1%)	30(46,1%)	**0.1**
Appui carpien ou pression sur le talon de la main	2(3%)	0	**0.23**
***Contraintes psychosociales***			
Demande psychologique	22,97 ± 2,26	23,73 ± 1,64	**0.29**
Latitude décisionnelle	55,5 ± 5,68	54,79 ±4,12	**0.19**
Soutien social	19,15 ± 1,80	18,12 ± 7,71	**0.69**
***Déclaration du SCC en MP***			**0.35**
***Reconnaissance du SCC en MP***	24(36,9%)	29(44,6%)	**0.13**

**Table 5:** Facteurs prédictifs du devenir professionnel des salariés opérés du SCC en analyse multi variée

	p	OR	IC
Qualification	0,05	0,12	[0,012-1,26]
Type de l’atteinte à l’EMG	0,05	0,55	[0,294-1,05]

## Discussion

Dans notre étude, la durée moyenne d’arrêt de travail pour les salariés opérés du canal carpien a été de 4 ±2.9 mois. Ceci concorde avec l’étude de Paris menée en 2004 auprès de 99 salariés opérés pour SCC d’origine professionnelle avec une durée moyenne d’arrêt de travail de 4 mois [5]. La durée moyenne d’arrêt de travail consécutif à l’intervention chirurgicale du SCC a été en moyenne de 1 mois et 6 jours avec des extrêmes allant de 0 à 38 mois d’après l’étude de Sillam et al menée en 2011 auprès de 969 patients [6]. Selon l’HAS, la durée d’arrêt de travail en post opératoire varie de 7 à 56 jours [7]. Une durée plus courte de 15 à 21 jours a été rapportée par Dreano et al. en 2011 [8]. Selon la littérature, l’arrêt de travail après traitement chirurgical du SCC dépend de plusieurs facteurs. De Kesela rapporté qu’une durée prolongée d’arrêt de travail pour les salariés opérés du canal carpien a été significativement liée au genre, au travail manuel avec une exposition à des tâches répétitives et à des vibrations [9]. Des résultats similaires ont été constatés dans notre série; 72,30 % des patientes ont repris leurs travails après 2 mois de l’acte chirurgical (9 semaines). Les patients exposés à des travaux qui sollicitent un appui carpien prolongé et un niveau élevé de flexion et d’extension des poignets ont une durée de repos postopératoire plus longue. Selon l’HAS, la durée d’arrêt de travail en post opératoire varie de 7 à 56 jours. Cette durée dépend de l’intensité du travail physique et de la modalité chirurgicale [7]. La durée d’arrêt de travail des opérés pour SCC dépend de la libération bilatérale du nerf médian, l´obésité, l’existence d’autres troubles musculo-squelettiques des membres supérieurs [10, 11]; ce qui se rapproche de nos résultats. En effet, nous avons noté une relation statistiquement significative de la durée d’arrêt de travail en post opératoire avec l’atteinte bilatérale et l’existence d’autre troubles musculo-squelettiques des membres supérieurs avec le SCC. En dehors des facteurs personnels et professionnels, le type d´assurance sociale des patients a été un facteur prédictif d’arrêt de travail en post opératoire [12]. Les patients couverts par les organismes de compensation des travailleurs ont un retour au travail beaucoup plus tard que ceux qui ont une assurance privée. Naglea décrit, dans une étude menée en 1994 auprès de 278 patients qui ont eu une libération chirurgicale endoscopique du canal carpien, une durée d’arrêt de travail moyenne de 65 jours pour les patients pris en charge par leur employeur, alors que pour les autres, la durée moyenne d’arrêt de travail n’a été que de 21 jours [13]. La déclaration du SCC en maladie professionnelle est un facteur de risque pour un arrêt prolongé du travail. Dans notre série, 73,84% des salariés ont bénéficié d’une reconnaissance de leur maladie en MP ; 60 % d’entre eux ont eu un arrêt de travail supérieur à 2 mois (9 semaines). Des constations similaires ont été aussi rapportées par Amick et al [14].

Dans notre étude, 50,7 % des patients ont gardé le même poste après l’intervention chirurgicale. 33.8% ont bénéficié d’un changement de poste dans la même entreprise et 15.3% un aménagement de poste. Ces résultats sont en concordance avec les données de la littérature. Bardouillet et al. ont noté dans leur étude sur le devenir médico-professionnel des opérés du canal carpien que 52.3% des salariés ont conservé le même poste de travail et 11.8 % ont bénéficié d’un aménagement de poste [15]. Yagev et al. ont trouvé qu’après intervention chirurgicale 50.2% des patients reprennent leur poste de travail alors que seulement 6% changent de poste [16]. Sillam et al. ont noté que 86% des personnes professionnellement actives au moment de leur consultation préopératoire ont été encore actives après six mois de l’intervention [6]. La plupart de ces personnes ont été au même poste de travail (95%) avec un aménagement de leurs conditions de travail pour 6 % d’entre elles. Paris M et al. ont trouvé que 69.7% des salariés ont conservé leur poste de travail après trois ans de l’intervention chirurgicale [5]. Dans notre étude, les difficultés du reclassement professionnel des victimes des troubles musculo-squelettiques d’origine professionnelle, en particulier le SCC semble être liés à deux facteurs principaux. : Le premier est en rapport avec la qualification professionnelle des victimes, dont la plupart sont des ouvriers d’un niveau éducationnel bas et pour lesquels une formation professionnelle serait difficile. Le deuxième était le type de l’atteinte sensitive/motrice à l’EMG.

En effet, le retour au travail peut être influencé par les conditions de travail plutôt que la situation médicale. Les interventions qui visent à améliorer le retour au travail après libération du canal carpien devront être multidimensionnelles. Dans la série de Pale et al., 73% des sujets opérés ont regagné leur poste de travail antérieur [17]. Bardouillet a conclu que les principaux facteurs qui influencent la reprise de travail sont: le type de prise en charge administrative de la pathologie, l’indice de la satisfaction, la notion de travail sous contrainte de temps [15]. Pour Schinkel les facteurs de mauvais pronostic ont été l’intervention simultanée sur un autre trouble musculo-squelettique du membre supérieur, la catégorie socioprofessionnelle « ouvriers » et l’arrêt de travail pour maladie professionnelle [18]. Katz JN et al. ont étudié les facteurs déterminants du devenir professionnel de 315 opérés pour SCC et suivis en post opératoire sur une période comprise entre 6 et 18 mois, ils ont rapporté que 45 % des patients ont bénéficié d’un changement du poste de travail. Selon la même étude, les facteurs prédictifs d’un changement de poste ont été le statut professionnel et la notion d’une indemnisation des salariés. Les autres prédicteurs moins puissants ont été le genre féminin et le secteur professionnel. Par contre l’âge, le niveau éducationnel et le type de traitement n’ont pas influencé le devenir professionnel des salariés opérés pour SCC [19]. En effet, une réexposition professionnelle aux contraintes professionnelles semble être un facteur de mauvais pronostic, aussi bien pour l’individu que pour l’entreprise, en termes d’altération de la qualité de vie, de baisse de la productivité et essentiellement en terme d’absentéisme. Dans ce sens, Katz JN. et alont conclu que les facteurs prédictifs d’absentéisme sont identiques à ceux du devenir professionnel des opérés pour SCC, et que l´état fonctionnel des travailleurs est le déterminant du retour au travail de ces patients et doit être la cible des actions qui visent le maintien de ces victimes au travail [19]. D’où la nécessité d’une collaboration des acteurs de prévention de santé et de sécurité au travail pour une réinsertion adaptée des opérés pour le SCC.

## Conclusion

Cette étude est parmi les rares études qui déterminent les critères objectifs qui peuvent influencer le retour au travail des salariés opérés pour SCC. En dépit de ces limites, il semble raisonnable de supposer que les résultats de cette enquête constituent le point de départ à des études plus larges et approfondies en particulier avec un suivi prospectif des patients victimes du SCC. Dans notre étude et en analyse multi variée, le devenir professionnel des salariés opérés a été corrélée avec la qualification professionnelle et au type de l’atteinte sensitive et/ou motrice du nerf médian à l’EMG. Le retour au travail peut être influencé par les conditions de travail plutôt que la situation médicale. Les interventions qui visent à améliorer le retour au travail après la libération du canal carpien doivent être multidimensionnelles. D’où la nécessité d’une collaboration des acteurs de la prévention de la santé et de la sécurité au travail pour une réinsertion adaptée des opérés pour SCC. Certes, il n’y a pas de solution universelle. Chaque entreprise doit élaborer sa propre démarche de prévention en fonction de l’analyse ergonomique.

### Etat des connaissances actuelle sur le sujet

- Plusieurs facteurs influencent la durée d’absence postopératoire du SCC;
- Le devenir professionnel des opérés pour SCC est peu étudié dans la littérature.

### Contribution de notre étude à la connaissance

- La durée d’absence postopératoire dépend essentiellement des facteurs liés à la vie professionnelle des salariés;
- Malgré le traitement chirurgical du SCC, la moitié des patients reste exposer aux mêmes facteurs de risque professionnel;
- Le reclassement professionnel des opérés pour SCC dépend des facteurs professionnel et médical.
